# Assessing the Growing Impact of Oral GLP-1 Agonists on National Obesity Policies of the United Kingdom

**DOI:** 10.7759/cureus.116274

**Published:** 2026-09-15

**Authors:** Alan Mathew, Hannah Anwar

**Affiliations:** 1 Internal Medicine, Basildon and Thurrock University Hospitals NHS Foundation Trust, Basildon, GBR; 2 Medical Education, Basildon and Thurrock University Hospitals NHS Foundation Trust, Basildon, GBR

**Keywords:** adverse effects of glp1 agonists, glp1 receptor agonist, healthcare access inequality, injectable glp-1 agonists, medical weight loss, medications adherence, national health service (nhs), obesity, oral semaglutide, public health policy

## Abstract

Obesity is a widely recognised major health concern within the United Kingdom (UK), culminating in rising costs for the National Health Service (NHS) per annum. Considering recent approval from regulatory bodies for the administration of oral glucagon-like peptide-1 (GLP-1) receptor agonists for weight management, this editorial explores the potential impact of oral GLP-1 receptor agonists on UK obesity policies, as well as establishing their safety profile and purported long-term health outcomes. It outlines key challenges faced by the NHS and the UK Government, such as growing inequalities in current access to the medication and the assurance of fair state provision, targeting the highest-risk patient groups first. Whilst national policy in tackling obesity is an ever-evolving landscape, it is imperative that oral GLP-1 receptor agonists are recognised by the wider populace not as substitutes for established methods but as tools to augment their efficacy.

## Editorial

In June 2026, the first oral glucagon-like peptide-1 (GLP-1) receptor agonist (semaglutide (Wegovy) titrated to 25 mg once daily) was approved for weight management in the United Kingdom (UK) after passing the Medicines and Healthcare products Regulatory Agency (MHRA) criteria for safety [1]. Recent statistics concluded that 14.3 million adults in England were classified as obese or severely obese [2], with the total financial cost of treatment by the NHS valued at £9.3 billion [3]. With the emergence of GLP-1 receptor agonists as a complementary measure alongside established dietary and lifestyle recommendations, there is growing concern from healthcare professionals that the government needs to streamline its policies with regard to long-term obesity prevention [4]. The efficacy of this medication class has been well established, with recent literature concluding that a higher proportion of patients achieved weight loss with GLP-1 receptor agonists (78.54%) compared to placebo (26.53%) [5]. Despite this, it is imperative that the UK government implements stricter regulation with regard to prescription and monitoring, given the surge in GLP-1 receptor agonist uptake in the adult population, with an estimated 2.4 million people in the UK with registered prescriptions [6], although ~90% of these individuals are acquiring them through the private sector [7]. The Department of Health and Social Care has explicitly stated that it aims to establish a wider NHS rollout so that patients in the highest risk groups can be administered the medication in a timely manner [1]. 

Injectable GLP-1 receptor agonists are already widely used for treatment of medical obesity, although they are limited by cost, compliance and the impracticality of administration [8]. Medications in tablet form, by contrast, are widely preferred to injectable forms due to convenience, non-invasive method of administration, and the potential for reduced cost. Clinical trials for GLP-1 receptor agonists have been undertaken, with two crucial phase-3 trials completed in September 2025 on oral semaglutide and orforglipron. These distinct trials compared the formulation of each tablet and investigated the effectiveness, side effects, and key safety concerns. Their results demonstrated effectiveness in both medications, with orforglipron producing "a mean weight loss of 11.2%, ≥10% weight loss in 54.6%" of participants over a 72-week period in 3,127 adults, compared with a "mean weight loss of 13.6%, ≥10% weight loss in 63%" for oral semaglutide over a 64-week period in 307 adults. Safety findings in these trials noted common GI side effects as associated with many oral medications, including nausea, vomiting, and diarrhoea, but highlighted more concerning safety issues including pancreatitis, tachycardia, and mild dysaesthesia [8]. It is important to note that semaglutide and orforglipron have two distinct mechanisms, with the former requiring an absorption enhancer, which directly impacts manufacturing scalability and food timing restrictions. However, more evidence is needed on long-term effectiveness, side effects, and safety concerns regarding oral GLP-1 receptor agonists. 

A recent systematic review and meta-analysis by West et al. surmised that patients who stopped weight management medications returned to baseline weight after 1.5-2 years, faster than the predicted timeline suggested by the National Institute for Health and Care Excellence (NICE) of two years [9]. Whilst there is a lack of long-term evidence surrounding health outcomes, early studies from Denmark in patients with type 2 diabetes showed that adherence rates were ~50% after one year of treatment, with a further 25% of patients discontinuing treatment (outside of clinical control trials) [10]. These figures directly correlated with lower socioeconomic status. Such findings are analogous to data provided by independent weight-loss management providers in the UK, which suggest that ~45% of individuals aged 30-49 commence treatment with BMI > 35 compared to ~30% in areas with increased socio-economic deprivation [6]. These data reflect glaring health inequalities in the current administration of oral GLP-1 receptor agonists across the country. It is therefore paramount that the NHS should aim to target patients in the highest risk groups. However, caution should be exercised with regard to overall long-term obesity policy. Patients should be explicitly informed that GLP-1 receptor agonists are not a substitute for sustained long-term lifestyle changes, which can be provided through schemes such as the NHS Digital Weight Management Programme, which saw an average reduction of 3.9 kg over 12 weeks (between April 2021 and March 2022) in 14,268 patients [11]. Further legislative incentives should be provided by the government to ensure the promotion of a healthier lifestyle to the adult population. 

The rapidly increasing prevalence of obesity in the worldwide population is driven by several factors and increases the demand for weight loss medications, with oral GLP-1 receptor agonists being advertised as the new forefront of weight loss medications. It puts pressure on pharmaceutical companies to provide safe, convenient, and effective medications, both in results and cost, that are widely available for public healthcare use. Oral forms of GLP-1 receptor agonists are presumed to increase adherence in comparison to injectable forms. However, there is already evidence to suggest that the convenience of oral GLP-1 receptor agonists does not directly correlate with adherence, as is already the issue with many oral medications. A wider rollout of oral GLP-1 receptor agonists prior to a large amount of data and research risks safety issues to individuals as well as worsening the pre-existing inequalities in medicine availability in more deprived areas. A consideration for a phased rollout of oral GLP-1 receptor agonists, where pharmacovigilance is utilised to track long-term side effects, should be taken, as the current MHRA approval is mainly based on short-term safety data. The convenience of oral GLP-1 receptor agonists should not outweigh the safety concerns, and the need for sufficient evidence to ensure that healthcare providers have confidence in prescribing oral GLP-1 receptor agonist tablets and users have confidence in the safety of taking these medications. 
